# Rewriting the history of leishmaniasis in Sri Lanka: An untold story since 1904

**DOI:** 10.1371/journal.pntd.0010918

**Published:** 2022-12-08

**Authors:** Hasara Nuwangi, Kosala Gayan Weerakoon, Thilini Chanchala Agampodi, Helen Philippa Price, Lisa Dikomitis, Suneth Buddhika Agampodi

**Affiliations:** 1 Department of Community Medicine, Faculty of Medicine and Allied Sciences, Rajarata University of Sri Lanka, Sri Lanka; 2 Department of Parasitology, Faculty of Medicine and Allied Sciences, Rajarata University of Sri Lanka, Sri Lanka; 3 Centre for Applied Entomology and Parasitology, School of Life Sciences, Keele University, Newcastle-under-Lyme, Staffordshire, United Kingdom; 4 Kent and Medway Medical School, University of Kent and Canterbury Christ Church University, Canterbury, United Kingdom; Centro de Pesquisa Gonçalo Moniz-FIOCRUZ/BA, BRAZIL

## Abstract

Leishmaniasis is widely considered a disease that emerged in Sri Lanka in the 1990s. However, a comprehensive case report from 1904 suggests that the presence of *Leishmaniasis* was well demonstrated in Sri Lanka long before that. The Annual Administration Reports of Ceylon/Sri Lanka from 1895 to 1970 and the Ceylon Blue Book from 1821 to 1937 are official historical documents that provide an annual performance, progress, goals achieved, and finances of Sri Lanka during that time. Both these documents are available in the National Archives. The Ceylon Administrative Report of 1904 reports a full record of observation of Leishman-Donovan bodies in Sri Lanka for the first time. These reports contain a total of 33,438 cases of leishmaniasis in the years 1928 to 1938, 1953, 1956, 1957, 1959, 1960, and 1961 to 1962. Up to 1938, the term “cutaneous leishmaniasis” was used, and after 1938, the term “leishmaniasis” was used in these reports. “Kala-azar” was also mentioned in 11 administrative reports between 1900 and 1947. In 1947, an extensive vector study has been carried out where they reported kala-azar cases. This well-documented government health information clearly shows that the history of leishmaniasis is almost the same as the global history in which the first case with Leishman-Donovan bodies were reported in 1903.

## Leishmaniasis in recent years in Sri Lanka

In 1992, Athukorala and colleagues reported a locally acquired case of cutaneous leishmaniasis (CL) from the southern province of Sri Lanka [1] and claimed it as the “first” such case, which has been reiterated by citations in many scientific papers. In a similar vein, a case of visceral leishmaniasis (VL) was reported in 2007, and this has more than 45 citations over 15 years as the first case of VL in Sri Lanka [2].

However, evidence from comparative genomic analysis [3,4] clearly suggested that the Sri Lankan strain of *Leishmania donovani* has branched out and evolved over a long period of time, indicating that CL is Sri Lanka is not a newly emerged disease. Contrary to the popular belief of leishmaniasis as a “newly emerged” disease, the first case report on leishmaniasis in Sri Lanka was published by Castellani back in 1904 [5]. However, only a handful of scientific papers have reported this observation as historic evidence of *L*. *donovani* transmission in Sri Lanka [4,6–9].

## The disease surveillance in the late 19th and early 20th centuries in Sri Lanka

The Civil Medical Department of Ceylon was established in 1859, in which the sanitary branch was established in 1915. Since 1867, the Governor, the head of the colonial administration, sent Annual Administration Reports to the Crown consisting of a compilation of reports from government agents and heads of the departments/public institutions. In the Annual Administration Reports from 1895 to 1951, a comprehensive account of the health status of the country was included under the section on medical matters. In addition to this detailed report, the Ceylon Blue Book also included summaries of health-related matters of Sri Lanka. The Ceylon Blue Book was published between 1821 and 1937 as an annual comprehensive statistical report on various activities carried out in the country. The Department of National Archives of Sri Lanka has all these documents, namely, Ceylon Administration Reports, Report of The Principal Civil Medical Officer and Inspector-General of Hospitals (1895 to 1924), Administration Report of The Director of Medical and Sanitary Services (1925 to 1951), and Administration Report of The Director of Health Services (1952 to 1970). A review of these records and the Ceylon Blue Book (1821 to 1937) clearly provides hard evidence to show the history of leishmaniasis in Sri Lanka is different from what has been reported recently.

## Historical cases of kala-azar and the detection of Leishman-Donovan bodies in Sri Lanka

In 1904, Leishman-Donovan bodies were recorded for the first time and we have found evidence in the archives of a laboratory-confirmed case of VL in Sri Lanka in the same year. In the report, the Director of the Bacteriological Institute, Dr. Aldo Castellani, gives a detailed description of the investigations under the subtopic “Parasites: Leishman-Donovan bodies.” He describes the routine investigations carried out on a deceased 20-year-old male who showed the symptoms of tropical splenomegaly. Castellani made smears from the spleen as a matter of routine work stained with the Leishman modification of Romanowsky’s method and reports that “the bodies were well stained and identical to the bodies I have seen in the preparations of Donovan, Manson, and Low.” This was the same case reported in the British Medical Journal in 1904 [5]. In the same report, the Principal Civil Medical Officer and Inspector-General of Hospital declared that “Leishman-Donovan bodies have been demonstrated in Ceylon, and that many cases of ancylostomiasis and of so-called malarial cachexia are very likely cases of kala-azar.” The same statement was repeated in the administrative reports in 1905.

In 1952, four blood smears were investigated by the Department of Parasitology for *Leishmania donovani*. In the same report, under the Clinical Pathology section, they mention that one blood sample has been tested for Donovan bodies (which was typed as “Denovon bodies” in the report). The following year, the Department of Parasitology reported that 13 blood tests had been conducted as part of routine examination done for diagnostic purposes (reported as blood films for “*Leishmania donovani*”), and the Parasitology Laboratory of the General Hospital, Colombo had conducted 18 blood tests for Leishman-Donovan bodies (reported as Donovan bodies Leishman).

Within these seventy-five records, kala-azar was mentioned in 1900, 1910 to 1916, 1936 to 1938, and in 1947 and 1948 when they conducted an extensive vector study. Between 1936 and 1938, the reports record zero deaths and zero cases of kala-azar. After 1938, kala-azar is not reported until 1947.

In 1947, an extensive entomological investigation was conducted on the Northern Sri Lankan island of Delft, upon finding high sandfly prevalence in the area. This investigation was done by the medical research institute and the medical entomologist in collaboration. The report states that there were five types of *Phlebotomus* species present and *Phlebotomus argentipes*, which is the carrier of kala-azar represented around 90% of the total catch. The 1947 report, under “section 08—Medical Research Institute,” presents a special account of the prevalence of kala-azar. They report that 21,772 specimens were subjected to the Formol-Gel test for kala-azar, and 41 of them gave a positive reaction. They then carried out a special investigation for kala-azar cases in Delft, Jaffna, and Hambantota, but all suspected cases were reported as negative. As of the 1948 report in section “VI–Medical Research Institute,” 59,343 aldehyde tests for kala-azar had been performed. But the number of kala-azar positives or negatives was not specified in the report.

## Historical cases of cutaneous leishmaniasis in Sri Lanka

CL was first included in the reports as “Cutaneous leishmaniasis” in 1928, and reporting continued consecutively for 10 years. After 1938, the cases were reported irregularly and described in 1953, 1956, 1957, 1959, 1960, and 1961 to 1962 as “leishmaniasis.” The reported number of cases and deaths in the relevant years where CL was reported are shown in Table 1. No other information was reported apart from the case numbers and numbers of deaths. Reported cases between 1928 and 1937 were cross-checked with the Ceylon Blue book, and there were no discrepancies.

**Table 1:** The records of cases and deaths of leishmaniasis in historic health administration reports of Sri Lanka between 1928–1962.

Name of the record	Term/s used in the report	Admissions	Deaths	Total cases treated within the year
Administration Report of The Director of Medical and Sanitary Services 1928	Cutaneous leishmaniasis	3,338	17	3,436
Administration Report of The Director of Medical and Sanitary Services 1929	Cutaneous leishmaniasis	3,991	4	4,171
Administration Report of The Director of Medical and Sanitary Services 1930	Cutaneous leishmaniasis	2,375	7	2,482
Administration Report of The Director of Medical and Sanitary Services 1931	Cutaneous leishmaniasis	1,802	23	1,877
Administration Report of The Director of Medical and Sanitary Services 1932	Cutaneous leishmaniasis	2,128	11	2,190
Administration Report of The Director of Medical and Sanitary Services 1933	Cutaneous leishmaniasis	2,564	16	2,653
Administration Report of The Director of Medical and Sanitary Services 1934	Cutaneous leishmaniasis	1,031	17	1,086
Administration Report of The Director of Medical and Sanitary Services 1935	Cutaneous leishmaniasis	930	12	966
Administration Report of The Director of Medical and Sanitary Services 1936	Cutaneous leishmaniasis	4,854	18	5,096
Administration Report of The Director of Medical and Sanitary Services 1937	Cutaneous leishmaniasis	4,060	23	1,222
Administration Report of The Director of Medical and Sanitary Services 1938	Cutaneous leishmaniasis	6,341	17	6,578
Administration Report of The Director of Health Services 1953	Leishmaniasis	3	-	No data
Administration Report of The Director of Health Services 1956	Leishmaniasis	2	-	No data
Administration Report of The Director of Health Services 1957	Leishmaniasis	-	-	No data
Administration Report of The Director of Health Services 1959	Leishmaniasis	-	-	No data
Administration Report of The Director of Health Services 1960	Leishmaniasis	-	-	No data
Administration Report of The Director of Health Services 1961–1962	“Leeshmeiniyawa” leishmaniasis	2	-	No data

The reports between 1895 and 1900 did not have any leishmaniasis-related content. None of the reports contained any records on mucocutaneous leishmaniasis and did not mention the term “visceral leishmaniasis.”

## Placing the local evidence in the global context

The first laboratory confirmation of leishmaniasis in Sri Lanka was indeed carried out in 1904, only one year after the first descriptions of the *Leishmania* parasite in human samples by Leishman and Ross, despite the claims in the scientific literature that leishmaniasis emerged in Sri Lanka in the early 1990s [5]. The present global history documents on leishmaniasis therefore require revisions with Sri Lanka pushing back in the timeline by almost nine decades.

It is also important to note that the documented history of leishmaniasis in Sri Lanka may extend well before the early 20th century. Another important skin disease, “The Parangi disease,” which devastated the country since the 1800s, is extensively recorded in the history of Ceylon. In 1881, Dr. Kynsey, the Principal Civil Medical Officer and Inspector-General of Hospitals of Sri Lanka (then known as Ceylon), drew a report on “the parangi disease” in which he claimed that the term could be an umbrella term to describe an array of cutaneous maladies such as yaws, scabies, tinea, Impetigo Ecthyma, leprosy, etc. [10]. In this report, the assistant colonial surgeon remarks that “the parangi disease” of Ceylon could be the same as “Delhi boil,” and in one of the annexed articles from Medical Time and Gazette, the report quotes that, “Delhi boil,” “Moultan sore,” “Aleppo evil,” “Biskra button,” “Yemen and Aden ulcers,” and “the parangi disease of Ceylon” could be the same disease [11]. These terms are used to describe CL, and some of these are still being used in different parts of the world [12,13]. This claim was reconfirmed by John Murray in 1883 after reviewing pictures of “the parangi disease” from the library of the college of physicians [14]. It is likely that CL was prevalent in Sri Lanka and was described and treated under “the parangi disease” even during the 1880s.

Identification of leishmaniasis cases in the early 1990s has led to (re)introducing the disease to the Sri Lankan disease surveillance system. Surveillance, clinical, and genetic evidence clearly show that leishmaniasis is not a disease that emerged in Sri Lanka in the early 1990s but rather a reemerging disease that affected thousands of people in the early part of the 20th century. The widespread use of dichlorodiphenyltrichloroethane (DDT) in malaria control and probable vector control may have impacted on the incidence of leishmaniasis in the 1950s, but this needs further investigation. Local and global references given to leishmaniasis in Sri Lanka as a newly emerging problem need to be updated urgently.

## Dislaimer

The views expressed in this article are those of the authors and not necessarily those of the NIHR or the UK Department of Health and Social Care.

## References

[1] Athukorale DN, Seneviratne JKK, Ihalamulla RL, Premaratne UN. Locally Acquired Cutaneous Leishmaniasis in Sri Lanka. J Trop Med Hyg. 1992;95:432–33. 1460704

[2] Abeygunasekara PH, Costa YJ, Seneviratne N, Ratnatunga N, Wijesundera M de S. Locally acquired visceral leishmaniasis in Sri Lanka. Ceylon Med J. 2007;52: 30–31. doi: 10.4038/cmj.v52i1.1047 17585579

[3] Karunaweera ND. Leishmania donovani causing cutaneous leishmaniasis in Sri Lanka: a wolf in sheep’s clothing? Trends Parasitol. 2009;25:458–63. doi: 10.1016/j.pt.2009.07.002 19734098

[4] Siriwardana Y, Deepachandi B, Weliange S de S, Udagedara C, Wickremarathne C, Warnasuriya W, et al. First Evidence for Two Independent and Different Leishmaniasis Transmission Foci in Sri Lanka: Recent Introduction or Long-Term Existence? J Trop Med. 2019;2019:1–11. doi: 10.1155/2019/6475939 31428163 PMC6683790

[5] Castellani A. “Leishmania Donovani” in Ceylon. Seventy-Second Annual Meeting of the British Medical Association, BMJ. 1904;2: 629–78. doi: 10.1136/bmj.2.2281.629

[6] Wijesundera MS. Cutaneous leishmaniasis: an emerging health risk in Sri Lanka. Ceylon Med J. 2001;46:151–52. doi: 10.4038/cmj.v46i4.6467 12164035

[7] Ranawaka RR, Weerakoon HS, de Silva SH. The quality of life of Sri Lankan patients with cutaneous leishmaniasis. Mymensingh Med J. 2014;23:345–51. 24858165

[8] Ranawaka RR, Weerakoon HS, de Silva SHP. Randomized, double-blind, controlled, comparative study on intralesional 10% and 15% hypertonic saline versus intralesional sodium stibogluconate in Leishmania donovani cutaneous leishmaniasis. Int J Dermatol. 2015;54:555–563. doi: 10.1111/ijd.12685 25600472

[9] Siriwardana HVYD, Thalagala N, Karunaweera ND. Clinical and epidemiological studies on the cutaneous leishmaniasis caused by Leishmania (Leishmania) donovani in Sri Lanka. Ann Trop Med Parasitol. 2010;104:213–23. doi: 10.1179/136485910X12647085215615 20507695

[10] Kinsey WR. The Parangi Disease of Ceylon. 1881.

[11] Milroy G. Yaws and some allied diseases. Medical times and Gazette. 1878.

[12] Steverding D. The history of leishmaniasis. Parasit Vectors. 2017;10:1–10. doi: 10.1186/s13071-017-2028-5 28202044 PMC5312593

[13] Nazzaro G, Rovaris M, Veraldi S. Leishmaniasis: A disease with many names. JAMA Dermatol. 2014;150:1204. doi: 10.1001/jamadermatol.2014.1015 25389793

[14] Murray J. The Delhi and Oriental Sore. Trans Epidemiol Soc Lond. 1883;2:90–98. 29419078 PMC5529853

